# IDENTIFYING NEW ADRD DIAGNOSES IN HOME HEALTH CARE PATIENTS USING NATURAL LANGUAGE PROCESSING OF NURSES’ NOTES

**DOI:** 10.1093/geroni/igad104.3406

**Published:** 2023-12-21

**Authors:** Yolanda Barrón, Miriam Ryvicker, Jiyoun Song, Maryam Zolnoori, Maxim Topaz

**Affiliations:** VNS Health, New York City, New York, United States; VNS Health, New York City, New York, United States; University of Pennsylvania School of Nursing, Philadelphia, Pennsylvania, United States; Columbia University, New York City, New York, United States; Columbia University, New York City, New York, United States

## Abstract

Patients at risk of developing Alzheimer’s Disease and Related Dementias (ADRD) admitted to home health care (HHC) may show signs of the disease before receiving a formal diagnosis. HHC nurses are uniquely positioned to observe signs of patients’ cognitive symptoms and may record them in free-text notes in the electronic health record (EHR). We applied natural language processing (NLP) to approximately 1.8 million nurses’ notes to identify signs and symptoms of ADRD in a retrospective cohort of 56,652 patients receiving care from a large HHC agency. In a subset of 38,032 patients starting HHC without an ADRD diagnosis, we used survival analysis competing risks models to examine associations between NLP-derived indicators of ADRD constructs during the HHC episode and new ADRD diagnoses during a four-year follow-up period, adjusting for patient demographics, clinical characteristics, and functional status derived from structured standard assessment tools. By the end of follow-up, 23% of the patients were newly diagnosed with ADRD, while 35% died without a diagnosis. We observed higher 4-year cumulative incidence rates of ADRD when more ADRD-related symptoms were present in the nurses’ notes (e.g., confusion only, 0.35, 95% CI: 0.32; 0.38, memory loss only, 0.38, 95% CI: 0.36, 0.39; both, 0.52, 95% CI: 0.48, 0.56; none, 0.10, 95% CI 0.097, 0.103). These findings indicate that data from free-text notes taken during HHC serve as significant predictors for potential ADRD diagnoses. Such information can facilitate prompt specialist referrals, allowing for early diagnosis and potentially more effective treatments during the disease’s initial stages.

